# Characterization of the Skin Cultivable Microbiota Composition of the Frog *Pelophylax perezi* Inhabiting Different Environments

**DOI:** 10.3390/ijerph18052585

**Published:** 2021-03-05

**Authors:** Diogo Neves Proença, Emanuele Fasola, Isabel Lopes, Paula V. Morais

**Affiliations:** 1 Department of Life Sciences and Centre for Mechanical Engineering, Materials and Processes, University of Coimbra, Calçada Martim de Freitas, 3000-456 Coimbra, Portugal; diogo.proenca@uc.pt; 2 CESAM and Department of Biology, University of Aveiro, 3810-005 Aveiro, Portugal; emanuele.fasola@ua.pt (E.F.); ilopes@ua.pt (I.L.)

**Keywords:** cutaneous cultivable microbiota, acid mine drainage, amphibians, Perez’s frog, *Acinetobacter*, exopolysaccharide

## Abstract

Microorganisms that live in association with amphibian skin can play important roles in protecting their host. Within the scenarios of global change, it is important to understand how environmental disturbances, namely, metal pollution, can affect this microbiota. The aim of this study is to recognize core bacteria in the skin cultivable microbiota of the Perez frog (*Pelophylax perezi*) that are preserved regardless of the environmental conditions in which the frogs live. The characterization of these isolates revealed characteristics that can support their contributions to the ability of frogs to use metal impacted environments. Frog’s skin swabs were collected from *P. perezi* populations that inhabit a metal-polluted site and three reference (non-metal polluted) sites. Bacterial strains were isolated, identified, and subjected to an acid mine drainage tolerance (AMD) test, collected upstream from a site heavily contaminated with metals, and tested to produce extracellular polymeric substances (exopolysaccharide, EPS). All frog populations had *Acinetobacter* in their cutaneous cultivable microbiota. Significant growth inhibition was observed in all bacterial isolates exposed to 75% of AMD. EPS production was considered a characteristic of several isolates. The data obtained is a preliminary step but crucial to sustain that the cultivable microbiota is a mechanism for protecting frogs against environmental contamination.

## 1. Introduction

Amphibians are a group of vertebrates that harbor a rich and diverse microbiome on their skin [1,2,3]. It has been shown that this cutaneous microbiota may play an important role as a complement to the innate immune system of amphibians, by acting as a physical barrier, through the production of antimicrobial substances or even by stimulating the host immune system [4,5]. Amphibians colonized all continents (except Antarctica) with more than 7000 species, known nowadays [6,7]. Conservation practices must recognize menaces to amphibians’ populations. Almost all amphibian species live both in aquatic and terrestrial habitats; changing its physiology during their life [8]. Therefore, amphibians are susceptible to many stressors and environmental perturbations [9]. For some decades amphibians are the most threatened group of vertebrates [6,9,10], indeed 40% of all their species are in decline and about 30% are “critically endangered”, “endangered”, or “vulnerable” in the International Union for Conservation of Nature (IUCN) red list [7]. Habitat destruction, climate change, diseases, allochthonous species invasion, and pollution, are the most important stressors that accounted for the amphibians’ decline [9,11,12,13]. In fact, similar to what occurs in other vertebrate classes, some of the bacteria found in frog’s skin act as symbionts, enhancing hosts tolerance to pathogens [3,14,15,16,17,18,19,20,21]. In the context of the global amphibians’ crisis, pathogens are one of the major threats to their natural populations [22,23,24,25,26]. The effectiveness of skin bacteria in protecting against pathogens has already been proved by both in vitro and in vivo studies [15,16,27,28]. As some examples, in vitro assays by Kruger et al. [27] reported that bacteria isolated from the skin of *Lithobates clamitans* could inhibit the growth of *Batrachochytrium dendrobatidis* (*Bd*). In addition to *Janthinobacterium lividum* [19,21,28,29,30], anti-*Bd* bacteria from genera *Pseudomonas*, *Serratia,* and *Acinetobacter* were identified and found in several individuals in natural populations [21,31]. Therefore, it is important to study amphibians’ skin cultivable microbiota composition, understand its variations (among species, life stage, environment, and year’s season), and characterize the organisms that compose it.

Several authors have proven that the microbiome of amphibians is species-specific [1,3,32]. These microbiomes are additionally influenced by seasonal shifts and the health status of the sampled individual [21,33]. The establishment of the microbial skin community in amphibians is mainly obtained on the vertical transmission of parental microbes, horizontal transmission (e.g., during mating), pool of microbes present in the external environment (both aquatic and terrestrial), and on the interaction between the host’s immune system/mucous composition with bacteria [21,29,34,35]. Beyond this, the skin microbial community can be influenced by several biotic and abiotic environmental factors like chemical contamination [4,33,35,36,37,38]. However, the research about the influence of pollution on the skin microbiome composition is starting to understand how these changes are achieved. As a consequence, this dysbiosis could lead to an increased sensitivity towards infections with pathogens. Hernández-Gómez and colleagues [39] observed that exposure to environmental stressors led to higher relative abundances of *Flavobacterium* and *Acinetobacter*.

Bacterial biofilms have been shown to sequester metals from the water through an extracellular polymer matrix, composed of exopolysaccharides (EPS), proteins, and nucleic acids [40,41]. In a previous work, authors tried to relate the ability of bacteria to chelate metals in their EPS with positive effects in anurans. The biofilm assays performed revealed that bacteria isolated associated with frogs exposed to arsenic waters (*Boana faber*, *Ololygon luizotavioi*, and *Rhinella crucifer*) had higher biofilm production [42].

In such a context, it becomes crucial to understand the factors structuring the amphibians’ skin microbial communities. This study focused on the recognition of the core cultivable bacterial organisms in the skin cultivable microbiota of the Perez’s frog (*Pelophylax perezi*) that are preserved independently of the environmental conditions and that may support frogs’ ability to inhabit metal impacted environments. In the frame of this study, it was analyzed the sex-specific influence of environmental metal contamination on the diversity of the skin cultivable microbiota of Perez’s frog. To our knowledge, only one study addressed the skin microbiome of *P. perezi* natural populations [36]. In that study, Costa et al. [36], compared the microbiome of *P. perezi* populations inhabiting reference and chemically impacted sites, from different climatic regions of Portugal: Mediterranean temperate with marine influence (reference and salinized sites) and Mediterranean temperate (metal impacted site). These authors found that frogs sampled at the metal impacted site resulted in lower number of cultivable strains and lower bacterial diversity, comparatively to the other sites. However, whether those differences could also be due to metal impacted population originated from a different climatic region was not taken into consideration. In the present study, it is intended to bring new insights, by sampling all the *P. perezi* populations at the same climatic region. The identification of the core microbiota in the skin of this species, may in the future promote the use of these core bacteria as pro-biotics to protect vulnerable populations against metal contamination.

## 2. Materials and Methods

### 2.1. Sampling Sites

Four populations of the anuran species *Pelophylax perezi* (López-Seoane, 1885) were sampled at four sites in Portugal, one metal contaminated and three reference sites. This species has a conservation status of least concern. It is endemic and very common in the Iberian Peninsula, being capable of colonizing a wide diversity of habitats, including anthropogenically-modified ones, which enables obtaining individuals from a broad type of reference and impacted sites. The samples from the contaminated site were labeled with the letter C while letter R samples from reference sites; in the same way, M indicates male frogs and F females. Three sampling sites were considered as reference (Barragem de Reguengos 1 (BR1): 37°51′38 N/ 8°13′56 W (samples 17M and 20M); Barragem de Reguengos 2 (BR2): 37°51′38 N/ 8°14′11 W (samples 22F and 23M); Lagoa do Cao (LC): 37°55′20 N/ 8°06′44 W) (samples 9F, 10M, 11M, and 12F). The metal-polluted site (Ribeira da Água Forte (AF): 37°57′31 N/ 8°14′02 W) (samples 1F, 2M, 3F, 4M, 5F, 6M, 7M, and 8F) is a stream located in the Aljustrel mining area, in the Iberian Pyrite Belt. The stream drains from the area of the old mining dumps and has for decades been receiving acid mine drainage (AMD) [43,44,45]. This site was considered metal-contaminated according to the water quality Portuguese legislation, which follows the European guidelines (Decree-Law 236/98).

A sample of surface water was collected at the AMD to perform toxicity assays with the bacterium species, collected from the skin of *P. perezi*, and assess their tolerance to metal contamination. The total metal concentrations in the AMD, obtained by ICP-MS according to [36], were 2300 µg/L Al, 24 µg/L As, 95,000 µg/L Ca, 5.2 µg/L Cd, 0.7 µg/L Cr, 226 µg/L Cu, 18,000 µg/L Fe, 16,000 µg/L K, 43,000 µg/L Mg, 174,000 µg/L Na, 36 µg/L Ni, 10 µg/L Pb, and 2100 µg/L Zn. Conductivity (µS/cm), pH, and dissolved oxygen (DO, mg/L) values were 4240 µS/cm, pH 2.1, and DO 11.5 mg/L, respectively.

### 2.2. Collection of Skin Cultivable Microbiota

Sixteen frogs were captured with a hand net, eight frogs from the polluted site (five males, three females), and eight frogs from the reference sites (four males, four females). The animals were handled with nitrile gloves, previously disinfected with ethanol (70%). Immediately before skin cultivable microbiota collection, each frog was rinsed abundantly (dorsal and ventral side) with sterile distilled water to ensure the collection of skin-associated microbes rather than water-associated transient microbes [46]. The skin cultivable microbiota was collected using sterile swabs that were scrubbed along the animal, following the protocol described by Brem et al. [47]. To standardize the procedure, each frog was swabbed five times along the length of the ventral and dorsal region, head, lateral region, the surface of thigh and legs. All the frogs were maintained closed in a bucket for a limited amount of time to prevent double sampling and then released into their habitat. Swabs were placed in a sterile 1.5 mL tube with 500 µL of Nutrient Broth medium (Merck Millipore, Germany), 10x diluted, and immediately placed on ice until lab arrival, where they were further processed according to Costa et al. [36] for bacteria isolation.

### 2.3. Bacterial Isolates and Phylogenetic Analysis

Isolates from all sixteen-frog skin cultivable microbiota samples were obtained. Therefore, swabs were washed with 500 μL of 1/10 NB medium followed by plating the suspension, induplicate, on non-selective R2A agar medium (Difco Laboratories, Detroit, USA) and incubation at 22 °C for 8 days. Colonies with different morphotypes, based on color, border, size, brightness, and texture [48], were randomly isolated. In total 166 isolates were obtained, which were sub-cultured and preserved in NB-15% glycerol (*v*/*v*) at −80 °C. The number of CFU obtained for male and female samples was then compared by using a Student *t* test.

The phylogeny of the isolates was analyzed via 16S rRNA gene analysis of extracted DNA from the isolates according to Nielsen et al. [49]. Briefly, the bacterial strains grown on R2A plates and its cell mass was obtained by sterile loop. Bacterial cells were resuspended in TES buffer (50 mM Tris, 5 mM EDTA, 2.5% sucrose, pH 8) containing 20 mg lysozyme ml^−1^ and incubated at 37 °C for 1 h. Lysis buffer followed by 7.5 M ammonium acetate were added, mixed, and incubated on ice for 10 min. Microtubes were centrifuged 15 min at 16,000× *g* and supernatant was recovered to a new tube. The extraction was then performed with chloroform/isoamyl alcohol (24:1 *v*/*v*), followed by precipitation with 2-propanol. It was washed with ethanol 70%, dried and resuspended in ddH_2_O. The PCR of 16S rRNA gene was performed according to Proença et al. [50] and the amplicons were sent for sequencing by Macrogen company. To describe diversity indices from the obtained isolates, PAST 2.08 software (Reference manual: Øyvind Hammer, Natural History Museum University of Oslo, Sweden) [51] was used to calculate the Shannon–Weaver index (H′) and Simpson index. The Shannon–Weaver diversity indexes were compared between sites and sex by using the Hutcheson’s t-test. Additionally, similarities among bacterial strains were analyzed using Primer 6.1.6 (PRIMER-E Ltd., 2006) by performing the following analyses: non-metrical multidimensional scaling (NMDS, resemblance matrix constructed with the Jaccard’s method), and a two-way similarity percentage analysis (SIMPER, using Bray–Curtis similarity method). Redundancy analysis (RDA) was performed to reveal relationships between frog’s sex, bacterial genera, and the presence of pollution using the software package CANOCO (version 4.5.1; Microcomputer Power, Ithaca, NY, USA). A Monte Carlo permutation test was performed (*p* < 0.001) to evaluate the statistical significance of the effects of the explanatory variables on the species composition of the samples [52]. Venn diagrams were constructed using the VENN DIAGRAMS (http://bioinformatics.psb.ugent.be/beg/tools/venn-diagrams). (assessed on 13 October 2020)

### 2.4. Tolerance of Bacterial Isolates to AMD

To test the tolerance against AMD of all isolated species, representative strains were used to determine growth rates in the presence of three AMD dilutions (25%, 50%, and 75%) compared to a control (distilled autoclaved water). Therefore, a colony from each strain was suspended in LB medium to an OD_600nm_ of 0.4 and incubated at 22 °C for 48 h together with an autoclaved solution of AMD in the three different dilutions. Optical densities were measured at 600 nm using a Jenway 6405 UV Spectrophotometer (Keison International Ltd., Essex, UK). The toxicity of pure AMD (100%) was not included into analysis for two reasons: (i) natural populations of Perez’ frogs never live in waters presenting such a high metal and hydrogen ion concentrations, making the test with pure AMD an unrealistic scenario; (ii) pure AMD concentration could not be achieved because the inoculum preparation of bacterial cells was necessarily prepared in non-contaminated liquid medium, therefore adding the inoculum to AMD it could never result in a 100% AMD concentration. The bacterial growth at the AMD dilution was compared with bacterial growth in the control by mean of a one-way analysis of variance followed by the multicomparison Dunnett’s test. All parameters’ distributions were previously checked for normality and homoscedasticity of variance by using the Kolmogorov–Smirnov and the Levene tests, respectively. All the statistical analysis was performed by suing the software Statistica for Windows 8.0 (StatSoft, Aurora, CO, USA).

### 2.5. Exopolysaccharide (EPS) Production by Core Cultivable Microbiota Bacteria

Strains from all isolated species belonging to the core cultivable microbiota genus *Acinetobacter* were screened for EPS production as previously reported [53] with some modifications. Briefly, bacterial active cultures were streaked on Tryptic Soy Agar, supplemented with 5% of sucrose as carbon source, and incubated at 30 °C for 3 days. Bacterial colonies presenting slimy surfaces were reported as EPS-producing [53]. To confirm it, the bacterial isolates were transferred to Tryptic Soy broth medium with 5% of sucrose as carbon source and incubated at 30 °C for 24 h at 160 rpm. Cells were removed by centrifugation (11,500 rpm, 15 min, 4 °C) and supernatant was recovered. EPS presented in the supernatant was precipitated by cold ethanol (95%) in proportion 1:3 (*v*/*v*). EPS was recovered by centrifugation, after discard the supernatant, and dried to determinate the yield by weight.

### 2.6. Nucleotide Sequence Accession Numbers

The 16S rRNA gene sequences of the bacterial isolates reported in this study were deposited at Genbank database, under the accession numbers according KY611613–KY611778.

### 2.7. Ethic Statement

This work was carried out under the approval for research ethics of Direcção Geral de Veterinária-DGAV (the Portuguese institution responsible for authorizing animal experimentation research). The certificate of approval is provided within this submission.

## 3. Results

### 3.1. Bacterial Isolates of the Skin Cultivable Microbiota of P. perezi

The number of cultivable skin bacteria did not differ for samples obtained from the contaminated and reference sites and ranged between 5.22 ± 4.11 and 5.21 ± 4.18 log CFUs per frog sampled, respectively. Overall, CFUs obtained from male were slightly lower (*p* = 0.3481), compared to female (from all sites), with an average (±standard deviation) of 4.66 ± 2.88 and 5.94 ± 4.84, respectively. In this study, a total of 166 bacterial isolates were obtained to determine the frogs’ skin cultivable microbiota composition and further understanding their tolerance to AMD and ability to produce polymers. All the strains belonging to different species (104) were tested for toxicity analysis, representing 18 strains from CM, 18 strains from CF, 33 strains from RM, and 35 strains from RF. Nine *Acinetobacter* strains from all the different *Acinetobacter* species detected were tested for EPS production.

The bacterial diversity was estimated by the Shannon–Weaver diversity index (H′) and Margalef index (D). The H′ indices were 4.29 for RM, 4.21 for RF; 3.46 for CM, and 3.11 for CF, while D indexes were 2.48, 2.51, 2.24, and 2.09, respectively. Both indices showed a higher diversity of bacteria on the skin of frogs from reference sites. However significant differences were only observed between CF/CM (*p* = 0.020) and CF/RF (*p* = 0.028).

**Figure 1:**
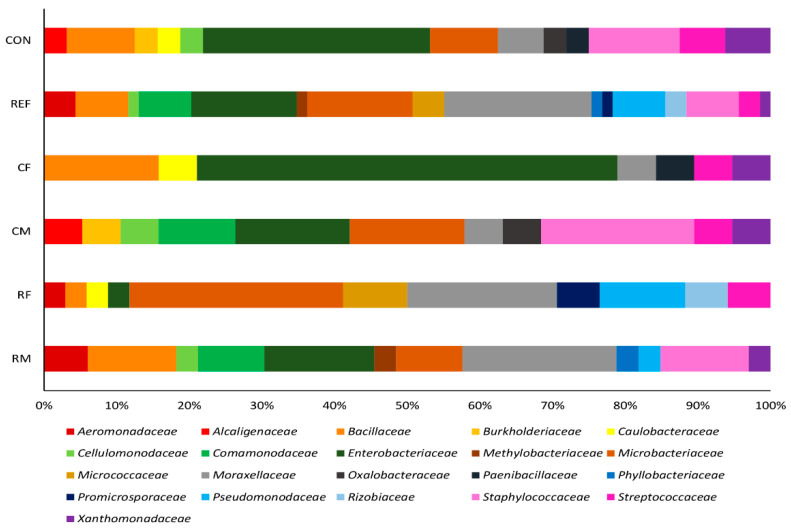
Cultivable microbiota composition of *Pelophylax perezi* skin at family level. The chart is divided into rows for (top to bottom): total samples from the metal-contaminated site (CON), total samples from the reference sites (REF), females (CF) and males (CM) frogs sampled at the metal-contaminated sites, and for female and male frogs sampled at the reference sites (RF and RM, respectively).

Phylogenetic analysis of the 16S rRNA gene sequences of the bacterial isolates, from all the sites, revealed that three phyla: Proteobacteria, Actinobacteria, and Firmicutes were dominating. Isolates belonging to *Proteobacteria* were dominant at all the sites, independently from contamination or frog’s sex (>60%), mostly belonging to Alphaproteobacteria, Betaproteobacteria, and Gammaproteobacteria. The most abundant families at the non-contaminated site were Enterobacteriaceae (20.3%), and Pseudomonadaceae (14.5%); while at the contaminated site, Enterobacteriaceae (31.3%) was most abundant followed by Staphylococcaceae (12.5%) was observed (Figure 1).

For the organisms sampled at the contaminated site, some differences were observed between sex; mainly for Enterobacteriaceae (CF—58%, CM—15.8%), Moraxellaceae (RF—20.6% and RM—21.2%), and Staphylococcaceae (CF—0%, CM—21.0%) (Figure 1).

**Figure 2:**
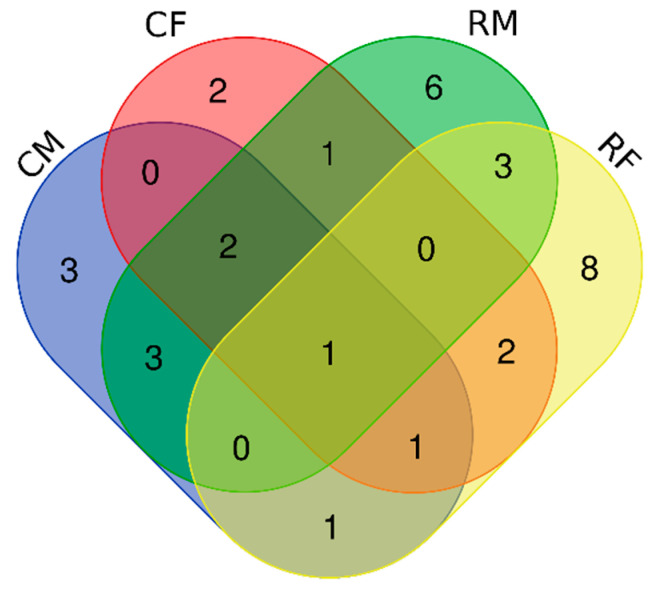
Venn diagram of the whole *Pelophylax perezi* skin microbial community at genus level. The central part consists in a core group, composed by bacteria belonging to the genera *Acinetobacter*; seventeen genera are present only in the reference sites while just five genera are restricted to the contaminated sites. CM, samples from contaminated sites of male frogs; CF, samples from contaminated sites of female frogs; RM, samples from reference sites of male frogs; RF, samples from reference sites of female frogs.

The frogs’ skin cultivable microbiota core group, here defined at genus level by bacteria present in all frogs’ samples, was composed of strains of the genus *Acinetobacter* (Figure 2). Five different bacterial genera were found only in samples from the contaminated site (*Paenibacillus*, *Erwinia*, *Herbaspirillum*, *Cupriavidus*, and *Bordetella*). Seventeen different genera were exclusively sampled in reference sites. Strains from *Staphylococcus*, *Cellulomonas,* and *Serratia* genera were present only on male frogs. On the other hand, female frogs shared exclusively strains from *Citrobacter* and *Brevundimonas* genera. The *Pseudomonas* strains cultivated in the present study were found only at reference sites, four on female and one on male frogs. Among the *Serratia* strains, four were found at the metal contaminated site and one at the reference site; all of them on male frogs.

**Figure 3:**
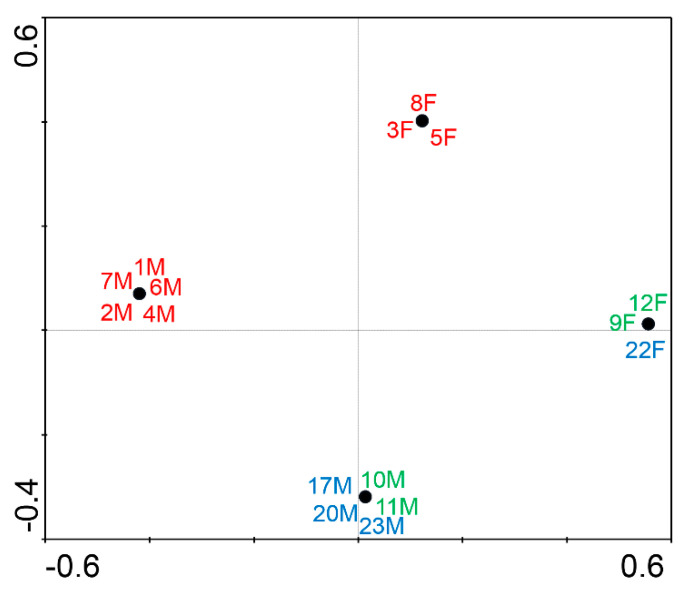
Redundancy analysis (RDA) of *Pelophylax perezi* skin bacterial samples, based in the differences between metal-contaminated and reference sites and between male and female frogs’ microbial communities. CF (3F, 5F, 8F), CM (1M, 2M, 4M, 6M, 7M), RF (9F, 12F, 22F), RM (10M, 11M, 17M, 20M, 23M). M = male frogs, F = female frogs. Red numbers indicate samples from AF (metal-contaminated) site, green numbers from BR sites and blue numbers from LC site, both reference sites.

The RDA analysis, for the cultivable microbiota fraction, supports a strong and clear distinction of the microbial community based on two factors: contamination of the sampling site and the sex of the frog (*p* = 0.014, Figure 3), but does not show a distinction between the microbial communities of the different reference sites.

### 3.2. Tolerance of Bacterial Isolates to AMD

**Figure 4:**
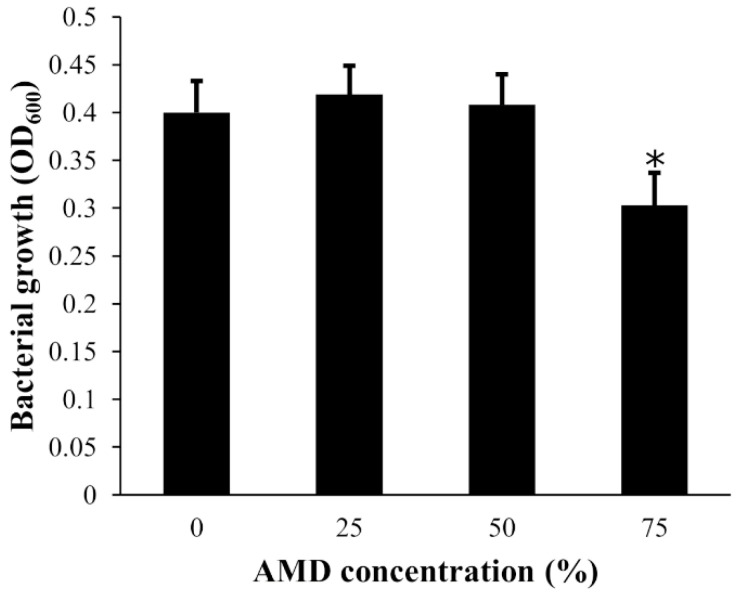
Average growth of *Pelophylax perezi* skin bacterial strains after being exposed to serial dilutions of acid mine drainage (AMD). Error bars represent standard deviation. * Statistically different from the control, *p* < 0.0001.

Statistical analysis revealed a significant inhibitory effect of the AMD, at dilution 75%, on the growth of all cultured bacterial strains (F_3800_ = 11.024, *p* < 0.0001), while no effect was detected at the other two tested dilutions (Figure 4). No significant differences in tolerance to AMD were detected between bacterial strains sampled in contaminated or reference sites (F_1800_ = 2.6944, *p* = 0.10110), which was confirmed by non-metric multidimensional scale analysis (NMDS, stress factor 0.01). All the bacteria strains grouped, except four outliers from the contaminated site, which belonged to the genera *Serratia, Stenotrophomonas, Phyllobacterium,* and *Erwinia*, all isolated from the skin of male frogs. This was confirmed by a SIMPER analysis where the same strains revealed a significant tolerance to AMD when compared to the others.

### 3.3. Exopolysaccharide (EPS) Production by Acinetobacter Strains

Nine *Acinetobacter* strains from the core cultivable microbiota genus were tested for EPS production. The strains selected belonged to all the different *Acinetobacter* species detected in the cultivable microbiota (Table 1).

The strains from the species Acinetobacter indicus, Acinetobacter junii, and Acinetobacter sp. 10M6B (Acinetobacter wuhouensis/Acinetobacter guillouiae) were considered positive for EPS production.

## 4. Discussion

The environment acts as a major driving force for host–microbe interactions [2,4,33,35,36]. In the present work, the composition of the skin cultivable microbiota of Perez’s frog was studied by cultivation methods. In addition, isolates were used to test the stress response of individual bacterial strains of the skin cultivable microbiota towards metal contamination. The ability to produce polysaccharides was also evaluated for the same group of organisms. It was possible to observe differences in the bacterial community composition, comparing contaminated and reference populations. The response pattern of cultivable microbiota differed in a sex-specific way. Differences in the composition of mucus’ peptides between male and female animals (in turn due to different hormonal inductions) [54,55] might explain this observation. Adding to this, males tend to be less philopatric moving among ponds to find a possible mate [56,57], which may potentiate their contact with more bacteria coming from different environments and other frogs (during mating).

The results of the present work are in line with other studies where the composition of amphibians’ skin cultivable microbiota showed to be influenced by the environment of the amphibians, the chemical and physical characteristics [58]. The present study unveiled that the richness of the skin cultivable microbiota of *P. perezi* living in reference environments is comparable with data from the previous study on the same frog species [36]. Moreover, it is in the same order of magnitude as that found for other amphibian species like *Rana italica* [59], a species related to the Perez’s frogs, and several other frog species [32]. The bacterial community in *P. perezi* skin samples was dominated by Proteobacteria, and Firmicutes, followed by Actinobacteria, as reported in previous studies on other amphibian species [2,32,36,59,60]. *Acinetobacter* was the most abundant genus, followed by *Staphylococcus*, *Microbacterium,* and *Bacillus*, in decreasing abundance. Of these four genus, *Acinetobacter* has been found abundant in ranid frogs’ skin microbiota [58].

The Shannon–Weaver diversity index was lower in frogs inhabiting the metal-contaminated site, compared to the ones inhabiting the reference sites. These results are in line with those obtained by Costa et al. [36], and, by studying populations from the same climatic region, goes further by confirming the influence of metal contamination in tailoring the amphibian skin cultivable microbiota. However, when comparing between RF and RM, males showed more diversity, in the analysis between CF and CM, females showed more diversity (between individuals from different sites). The obtained patterns suggest an ecological difference between AMD contaminated and reference sites. Which was marked in females, when comparing different sites. This can again be consistent with what was mentioned above regarding males being less philopatric, more movements among different sites can make their skin microbiota less site specific (less differences in male’s microbiota between individuals sampled in different sites) [56,57].

The toxicity assays proved that the growth of isolated bacterial strains was significantly reduced when exposed to 75% AMD, while no effects were detected at lower AMD concentrations. Even so, the NMDS analysis resulted in a compact group formation, leading to the fact that (1) microbial communities from all sampling sites harbor both sensitive and tolerant strains to AMD; and (2) all strains being able to grow, at least at 50% AMD, show a possible intrinsic capacity to tolerate metal-contaminated waters. The SIMPER analysis helped to identify four strains, which significantly contributed to the observed cultivable microbiota tolerance to AMD. These strains were *Erwinia* sp., *Serratia* sp., and *Stenotrophomonas* sp., which are commonly found associated with plants or fishes [61,62], and *Phyllobacterium* sp., which is a high yield exopolysaccharide producer. Furthermore, *Phyllobacterium*’s polymer has moistening preserving properties, which is a relevant property considering that the good function of amphibian’s skin (e.g., for dermal respiration) is highly dependent on its moistening level [63]. The present work is the first reporting that such bacterial species are part of the skin cultivable microbiota of amphibians.

This study confirms that the amphibians’ skin microbial community is influenced by metal pollution, but it is also determined by some sex-specific response pattern (at least in Perez’s frog). Moreover, the higher biofilm production could act as complementary protection against the entry of contaminants through the skin. This is an important frog skin protection since water acts as an important carrier of metals. Considering that, EPS production is an adaptive response of microorganisms when exposed to environmental stresses [64], the presence of metals in water (AMD-like) can act as stress inducer and directly affect polymers production. Additionally, the increase in EPS production may be a bacterial strategy to prevent their leaching from the frogs’ skin.

The core cultivable microbiota was composed of different species of the *Acinetobacter* genus. Four *Acinetobacter* strains from different species resisted 75% of AMD and two of them produced a high yield of EPS. Among the mechanisms of detoxification recognized in bacteria, the sequestration of extracellular (biosorption) and intracellular (bioaccumulation) metals [65,66], and the production and secretion of chelating molecules such as siderophores [67] are considered efficient. A *Serratia* strain was found among the ones particularly resistant to AMD exposure. Our research group showed that *Serratia* strains isolated from mine environments can express additional superoxide dismutases to control the cell oxidative stress, increasing strains metal resistance [68]. Furthermore, *Serratia* strains are known for their ability to form biofilm [69]. Here, it is foreseen that the most applicable bacterial mechanism that can act as a protective barrier to frogs’ skin could be related to bacteria biofilm production. Biofilm formed by EPS could have metal chelation function and adhesive properties.

## 5. Conclusions

The data presented in this work highlight that the Perez frogs’ skin cultivable microbiota core group was composed of strains of the genus *Acinetobacter*. Moreover, it was shown that the cultivable microbiota structure of the frogs’ skin was influenced by the physicochemical characteristics of the environment, but also determined by some sex-specific response patterns (at least in Perez’s frog). The metal tolerance and EPS production ability of the cultivable microbiota’s bacterial strains are a clear indication that the cultivable microbiota is a protection mechanism for frogs against environmental contamination.

The obtained data could be a preliminary but crucial step in understanding how skin cultivable microbiota works in amphibians and how it can be related to the frogs’ protection capabilities. The impact of changes in the cultivable microbiota and its functional characteristics on endangered species, whether threatened by a different kind of pollution or impacted by pathogens, is still to be discovered.

## Figures and Tables

**Table 1:** Core cultivable microbiota strains producing exopolysaccharide (EPS).

Strain	Bacterial Species	Identity (%)	Resistance to [AMD] (%)	Growth on TSB + 5% Sucrose (OD_600nm_)	EPS Production (g)	EPS.OD^−1^ (g)
22F13B	*Acinetobacter antiviralis KNF2022^T^*	97.6	75	1.01	0.05	0.05
12F14	*Acinetobacter beijerinckii NIPH 838^T^*	98.7	50	1.05	0.03	0.03
9F7	*Acinetobacter indicus A648^T^*	98.9	50	1.2	0.34	0.28
11M7	*Acinetobacter johnsonii DSM 6963^T^*	99.8	25	1.5	0.03	0.02
23M19	*Acinetobacter junii DSM 6964^T^*	100	75	1.09	0.45	0.41
10M2B	*Acinetobacter oryzae B23^T^/Acinetobacter johnsonii DSM 6963^T^*	98.6	50	1.28	0.05	0.04
8F3	*Acinetobacter pragensis ANC 4149^T^/Acinetobacter bohemicus ANC 3994^T^*	100	75	3.02	0.15	0.05
6M5	*Acinetobacter soli B1^T^*	99.2	25	2.27	0.11	0.05
10M6B	*Acinetobacter wuhouensis WCHA60^T^/Acinetobacter guillouiae DSM 590^T^*	98.4	75	1.17	0.19	0.16

## Data Availability

The 16S rRNA gene sequences of the bacterial isolates reported in this study were deposited at Genbank database, under the accession numbers according KY611613–KY611778.
